# Management of ART and COVID-19: Infertility in times of pandemic. What now?

**DOI:** 10.5935/1518-0557.20200031

**Published:** 2020

**Authors:** Maria do Carmo Borges de Souza, Hitomi Nakagawa, Paulo Franco Taitson, Emerson Barchi Cordts, Roberto Azevedo Antunes

**Affiliations:** 1 Latin American Network of Assisted Reproduction - REDLARA; 2 Brazilian Society of Assisted Reproduction - SBRA; 3 Fertipraxis Centro de Reprodução Humana - Rio de Janeiro - RJ, Brazil; 4 GENESIS – Center for Assistance in Human Reproduction, Brasília, DF, Brazil; 5 Pontifical Catholic University of Minas Gerais, Belo Horizonte, MG, Brazil; 6 Embryo Genesis Reprodução Humana e Instituto Ideia Fertil, Santo André, SP, Brazil

In January 30 of this year, for the sixth time in its history, World Health Organization (WHO) decreed a Public Health Emergency of International Importance - the Organization’s highest alert level. From then on, the infection due to the new Coronavirus, called COVID-19, was officially an international emergency (WHO, 2020a). On March 11, 2020, the situation escalated even more, as WHO declared that COVID-19 became pandemic due to its global geographical distribution (Monteleone *et al*., 2020).

Since then, several guidelines, epidemiological bulletins and letters of recommendations have been published and updated. In our area of expertise, Reproductive Medicine, the main medical societies in the field (ESHRE, ASRM, IFFS, REDLARA-SBRA) suggested that patients with infertility should consider deferring pregnancy (ASRM, 2020a; 2020b; ESHRE, 2020; IFFS, 2020; REDLARA-SBRA, 2020). Other common points suggested between fertility societies worldwide were to suspend initiation of reproductive treatments, including ovulation induction, intrauterine inseminations (IUIs), in vitro-fertilization, oocyte and sperm cryopreservation, as well as fresh/frozen embryo transfers. However, every organization highlighted that certain cases in which women were undergoing fertility preservation due to oncological reasons, as well as individual situations where postponing treatment could be more harmful than proceeding with it (ie: low ovarian reserve patients), should be performed if the center understood that it had conditions to be functional.

Reasons for undertaking these extraordinary steps included avoiding possible complications of assisted reproduction treatment and pregnancy that could contribute to the overflow of medical emergencies; lack of data regarding potential virus-related complications of pregnancy and concerns about vertical transmission to the fetus in COVID-19 positive patients. Additionally, this would contribute to the efforts for reallocation of critical healthcare personal and resources to aid in the COVID-19 patients aid. Finally, those measures would be consistent with current recommendations for social distancing. However, since the speed of dissemination of the COVID-19 worldwide took all fertility centers by surprise, and all the above-mentioned recommendations were published in an urgent fashion, globally (and Latin America was not different) all societies advocated that initiated cycles were to be completed and freeze all policy to be adopted, while transfers in preparation were discussed and mostly postponed.

Telemedicine and psychological support established a good tool to ART specialists. Several digital platforms quickly spread as valuable in favoring the communication and relationship between patients and physicians. Most fertility organizations expressly recommended the use of such tools during the pandemic.

Nonetheless, new data on COVID-19 continue to emerge and as research increases, new realities present them- selves in different countries, regions, states or cities. Other than that an extended period of pandemic is to be expected. Another important point of consideration in defining the directions of ART treatments regards two WHO key concepts: first infertility is a disease, and should be viewed as such. Second, all women should have autonomy to consider their family planning even in times of health-care crisis (WHO, 2019). Therefore, individualized recommendations, taking under consideration such geopolitical scenarios are urgently required.

So far, there is no clear difference comparing to non-pregnant and pregnant women that present COVID-19. They will both, most likely, experience mild/moderate symptoms, unless they are included in risk groups like hypertension, diabetes, etc. Recently puerperal women became a risk group as well (Ministério da Saúde- Brazil, 2020). Most COVID-19 pregnancies followed in China were >20 weeks and, so far, checking born babies, there is no clear evidence of vertical transmission of SARS- through COVID-19. However, data on the effects of the COVID-19 in the first trimester of pregnancy are still lacking. Therefore, a freeze all embryo policy, as well as, individualized embryo transfers only, should still be a recommendation. All the Healthcare facilities are aware of the main principles of care, especially those related to COVID-19 spread conditions, and what this means to both their teams and patients. At the same time, there is a huge pression from infertility patients overall. It is important to remember that the average age of couple trying to conceive is augmenting on a yearly basis, and that reflects on older patients, with worse ovarian reserve markers arriving on fertility centers. With the extended prevision of the COVID-19 pandemic emergency, as well as, the current recommendations to suspend ART treatments, most patients are anxious and scared with the real possibility of compromising even further their chances of pregnancy. The great dilemma to fertility centers is to establish safe conditions to be back to support couples without confronting local government regulations.

On April 7th, REDLARA and SBRA held an online event on Bioethics in the days of COVID-19, where Brazilian law authorities, bioethics specialists and ART professionals made it clear that medical judgment should prevail in the individualization of infertility cases, as well as patient autonomy. Therefore, individualization of each treatment is the cornerstone of the moment. ART treatments could be performed as long as they are well documented regarding the potential hazard of its delay, the informative consent signed by the patient agreeing to go through with the treatment during the pandemic state and providing access to freezing procedures. Good judgement is essential to allow ART clinics to keep caring for both their staff, and their patients.
